# Bayesian Optimization of Wet-Impregnated Co-Mo/Al_2_O_3_ Catalyst for Maximizing the Yield of Carbon Nanotube Synthesis

**DOI:** 10.3390/nano14010075

**Published:** 2023-12-26

**Authors:** Sangsoo Shin, Hyeongyun Song, Yeon Su Shin, Jaegeun Lee, Tae Hoon Seo

**Affiliations:** 1 School of Chemical Engineering, Pusan National University, Busan 46241, Republic of Korea; shinsangsu15@gmail.com (S.S.); shy0753@gmail.com (H.S.); syeonsu1212@gmail.com (Y.S.S.); 2 Department of Organic Material Science and Engineering, Pusan National University, Busan 46241, Republic of Korea; 3 Green Energy and Nano Technology & R&D Group, Korea Institute of Industrial Technology (KITECH), Gwangju 61012, Republic of Korea

**Keywords:** carbon nanotube, Bayesian optimization, wet-impregnation, catalyst, chemical vapor deposition

## Abstract

Multimetallic catalysts have demonstrated their high potential for the controlled synthesis of carbon nanotubes (CNTs), but their development requires a more complicated optimization than that of monometallic catalysts. Here, we employed Bayesian optimization (BO) to optimize the preparation of Co-Mo/Al_2_O_3_ catalyst using wet impregnation, with the goal of maximizing carbon yield in the chemical vapor deposition (CVD) synthesis of CNTs. In the catalyst preparation process, we selected four parameters to optimize: the weight percentage of metal, the ratio of Co to Mo in the catalyst, the drying temperature, and the calcination temperature. We ran two parallel BO processes to compare the performance of two types of acquisitions: expected improvement (EI), which does not consider noise, and one-shot knowledge gradient (OKG), which takes noise into account. As a result, both acquisition functions successfully optimized the carbon yield with similar performance. The result suggests that the use of EI, which has a lower computational load, is acceptable if the system has sufficient robustness. The investigation of the contour plots showed that the addition of Mo has a negative effect on carbon yield.

## 1. Introduction

Carbon nanotubes (CNTs) have become a significant component in various fields due to their outstanding electrical [1], thermal [2], and mechanical properties [3,4]. For example, the excellent mechanical properties of CNTs are realized on a macroscopic scale by assembling CNTs into CNT fiber [5,6,7,8]. Due to the high electric conductivity and one-dimensional nanostructure, CNTs are actively utilized as conductive additives in batteries [9]. Their electronic properties enable their application into field-effect transistors [10].

To meet the specific needs of different applications, it is necessary to synthesize CNTs with controlled properties. Catalytic chemical vapor deposition (CVD) is the most widely used method to synthesize CNTs. There are various CVD systems that are capable of producing CNTs. The primary distinction among these systems lies in the method of supplying catalyst nanoparticles. For instance, if the catalyst is formed from a thin metal film deposited on a flat surface, it is possible to grow vertically aligned CNT arrays, also known as CNT forests [11]. Catalyst nanoparticles can be loaded on porous support materials using several methods, including wet and dry impregnation [12,13]. Alternatively, catalyst precursors can be introduced directly into the CVD reactor to form catalyst nanoparticles in situ [14,15]. This technique is referred to as floating catalyst CVD.

Among various catalyst types, catalysts loaded on porous support are considered promising for controlling the nanostructure and enabling large-scale production [16,17,18,19,20]. The properties of CNTs are mainly determined by the catalyst, so many studies have focused on optimizing catalysts [21,22].

A common catalyst configuration for the synthesis of CNTs is monometallic nanoparticles loaded on ceramic support [23,24,25,26]. However, a bimetallic catalyst provides greater control over the synthesis of CNTs. The composition of a bimetallic catalyst considerably affects CNT growth, and several combinations have been explored. For instance, Co-Mo catalysts have shown the ability to control the selectivity toward single-walled CNTs (SWCNTs) [27]. Fe-Ni catalysts have a high catalytic activity for CNT growth [28]. Ti-Co catalysts enhance the growth of multi-walled CNTs [29]. Co-W catalysts enable the chirality-specific growth of SWCNTs [30]. Fe-Cu catalysts can adjust the chirality distribution of SWCNTs [31]. A Co-Cu catalyst can synthesize subnanometer-diameter SWCNTs [32].

The development of multimetallic catalysts requires a more complicated optimization than that of monometallic catalysts. As the number of catalyst components increases, the optimization of multimetallic catalysts has additional input parameters. Thus, the optimization of multimetallic catalysts requires more experiments than that of monometallic catalysts to complete the optimization. To address this challenge, we employed Bayesian optimization (BO) to optimize a bimetallic catalyst for the CVD growth of CNTs.

BO is a machine learning process based on Bayes’ theorem that can efficiently optimize a black-box function that is expensive or difficult to evaluate [33,34]. BO consists of two main components: a surrogate model and an acquisition function. The surrogate model is a probabilistic model that estimates the shape of a black-box function based on observed input-output values ((x_1_, f(x_1_), …, (x_n_, f(x_n_)). A Gaussian process is the most widely used model as the surrogate model [35]. It consists of a mean function and a covariance function. These functions are responsible for determining the expected value and the variability of the black-box function at any given point. The acquisition function recommends input values for the next experiment based on the probabilistic estimation of the surrogate model. This function balances the trade-off between exploration and exploitation. Exploration means selecting the next point in the input space where the uncertainty about the black-box function is high, while exploitation means choosing the next point that is close to the current best point. The balance between exploration and exploitation is crucial for the success of the optimization processes. Compared to classical optimization methods like design of experiments (DOE), BO offers several advantages. It minimizes the number of function evaluations by leveraging prior information and utilizing the structure of the function. In addition, it can handle noisy data by using robust priors and posterior distributions. These advantages make BO particularly effective in optimizing the catalyst preparation process to maximize the CNT yield.

Here, we employed BO to experimentally optimize the catalyst preparation process of Co-Mo/Al_2_O_3_ by wet impregnation, with the goal of maximizing carbon yield in a CVD synthesis of CNTs. We optimized four parameters of the catalyst preparation process. We also compared the performance of two types of acquisition functions, one that considers noise and one that does not, by conducting two parallel BO processes. Analysis of various response surfaces provides insight into the effect of the parameters on carbon yield.

## 2. Materials and Methods

### 2.1. Catalyst Preparation

The Co-Mo/Al_2_O_3_ catalysts were prepared using the wet impregnation method. Cobalt nitrate hexahydrate (98.0%, Matsugaki chemical industries, Oskak, Japan), ammonium heptamolybdate tetrahydrate (98%, Daejung chemical and metals, Siheung-si, Republic of Korea), and porous Al_2_O_3_ powder (99%, 32–63 μm SABET: 200 m^2^/g, Alfa Aesar, Haverhill, MA, USA) were stirred in an aqueous solution for 1 h at room temperature. The catalyst’s metal weight percent and Co: Mo metal loading ratio were chosen as recommended by BO. This mixture was then dried at a temperature recommended by BO while stirring. The sample was ground and then calcined in a horizontal furnace in an air atmosphere for two hours at a temperature recommended by the BO.

### 2.2. CNT Synthesis

CNT synthesis was performed in a horizontal furnace using a quartz tube with an inner diameter of 5.5 cm and a length of 1.3 m. For each run, we used 0.01 g of Co-Mo/Al_2_O_3_ catalysts to synthesize CNTs. The growth was run by a programmed recipe using a computer to precisely control experimental conditions and eliminate human error. N_2_ gas was used as an inert gas, and C_2_H_4_ gas was used as a carbon precursor. The synthesis was carried out at 690 °C with a flow rate of 30 sccm for C_2_H_4_, 30 sccm for H_2_, and 150 sccm for N_2_ gas for 10 min. The carbon yield was obtained from the following equation:

(1) $$Carbon\;yield\;(\%)=\left[\frac{M_{f}-M_{cat}}{M_{cat}}\right]\times 100$$

where *M_f_* refers to the mass of the final product containing the carbon product and Co-Mo/Al_2_O_3_ catalyst after the reaction, and *M_cat_* refers to the mass of the Co-Mo/Al_2_O_3_ catalyst used.

### 2.3. Bayesian Optimization

**Figure 1:**
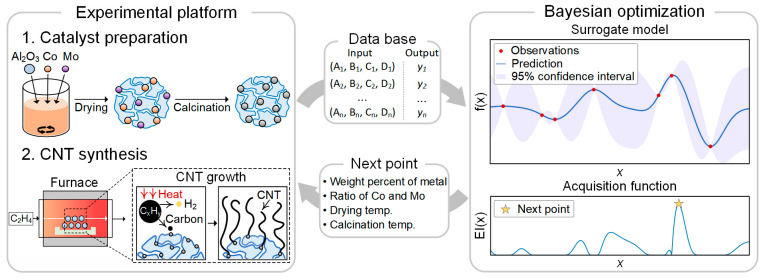
A schematic illustration of the BO process in this work.

We selected four parameters in the catalyst preparation process: weight percent of metal, ratio of Co and Mo in the catalyst, drying temperature, and calcination temperature. We set the range of four parameters: 1–70 wt.% for the weight percent of the metal, 80–300 °C for drying temperature, and 300–950 °C for calcination temperature. The ranges were decided experimentally from the physical constraints of the system. These parameters were optimized using BO to maximize the carbon yield in CNT synthesis. We used the Matern 5/2 kernel function for the Gaussian process and two types of acquisition functions: expected improvement (EI) and one-shot knowledge gradient (OKG) [36]. The BO process is schematically explained in Figure 1. First, a training database is constructed through experiments. This database is then fed into the BO algorithm, which generates a set of input parameters to investigate next. An experiment is conducted using the generated input parameters, and the results are added to the database. This process is repeated to optimize the parameters.

### 2.4. Characterization

The as-synthesized CNT samples were characterized using scanning electron microscopy (SEM), transmission electron microscopy (TEM), Raman spectroscopy, and thermogravimetric analysis (TGA). The morphology of CNT samples was observed using SEM (SUPRA 25, Carl Zeiss AG, Oberkochen, Germany). The diameter and wall number of CNT samples were measured by TEM (FEI-Titan Cubed 60–300 with a Cs-Corrector and monochromator at 80 kV). The *I_G_*/*I_D_* of the CNT samples was measured using a Raman spectrometer (NS240-F, Nanoscope Systems, Daejeon, Republic of Korea) with a laser excitation wavelength of 532 nm. We performed Raman measurements five times per sample. The purity of the CNT samples was investigated using TGA (Discovery TGA 55, TA Instruments, New Castle, DE, USA). The TGA analysis was conducted in air up to 900 °C at a heating rate of 10 °C/min.

## 3. Results and Discussion

### 3.1. Building the Initial Database

An initial database was constructed by examining 13 points, with four repeated experiments performed at each point, as detailed in Table 1. These points were recommended using a Sobol sequence, which is a type of quasi-random number generator that generates evenly distributed points within the input parameter space. Sobol sequence takes into account previously generated points and avoids the formation of clusters. The use of the Sobol sequence is known to be more effective in building an initial database [37]. The maximum carbon yield in the initial database was 244%.

### 3.2. Parallel Bayesian Optimization Processes

**Figure 2:**
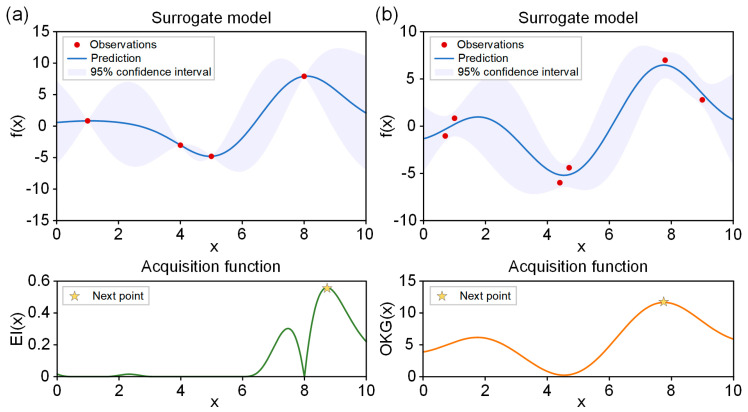
Illustration of surrogate model and acquisition function when using (**a**) EI (noise-free) and (**b**) OKG (with noise). The observations show previously investigated points (x_i_, f(x_i_)). The blue solid line is an estimate of the black-box function based on investigated points. The light blue is a 95% confidence interval.

When using BO, the selection of an appropriate acquisition function is crucial for successful optimization. There are several types of acquisition functions, such as probability of improvement, upper confidence bound, entropy search, EI, OKG, etc. When choosing an acquisition function, one important factor is whether to account for noise in the data. In fact, optimization of experimental parameters inevitably involves noise in the data. However, taking noise into account in the acquisition function incurs a computational cost. Here, we focused on the EI and OKG. The EI is the most commonly used acquisition function and recommends the next point without considering noise, meaning batch-to-batch variability (Figure 2a). In contrast, the OKG recommends the next point while taking noise into account (Figure 2b).

**Figure 3:**
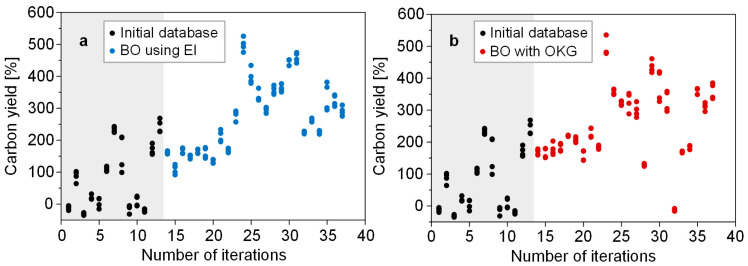
Carbon yield obtained from the two BO processes performed using (**a**) EI and (**b**) OKG as acquisition functions.

We performed two parallel BO processes using the EI and OKG as acquisition functions and compared their performance. After building the initial database, we carried out 24 iterations for each BO process, with four repeated experiments per iteration. Both BO processes successfully optimized the carbon yield (Figure 3). The detailed input and output variables obtained from the BO processes are listed in Table 2 and Table 3. The maximum carbon yield was 499% in the 11th iteration when using EI and 493% in the 10th iteration when using OKG. These maximum carbon yields observed in our study are notably higher compared to those reported in other studies that synthesized CNTs using a Co-Mo catalyst. For an accurate comparison of the yield, it is essential to consider the same growth time. In our study, the growth time was 10 min, and hence, we compared the carbon yield for this duration. Esteves et al. reported a carbon yield of 116% [38]. Aboul-Enein showed a carbon yield of 173% [39]. Yardimci exhibited a carbon yield of 254% [40]. Kludpantanapan reported a carbon yield of 10% [41]. Kibria showed a carbon yield of 253% [42].

The comparable performance of the BO can be attributed to the high robustness of our CNTs synthesis system [43]. A robust CNT synthesis system is one that can consistently synthesize CNTs of similar yield and quality, regardless of uncontrollable ambient laboratory conditions such as humidity and temperature. It is noteworthy that EI showed comparable performance to OKG, albeit at a significantly lower computational cost. This suggests that in a robust system, using an acquisition function that does not account for data noise could be a strategy to reduce unnecessary computational costs. Therefore, we suggest that EI is a viable option if the system exhibits sufficient robustness.

The high carbon yield at the optimum synthesis implies that the Co nanoparticles are evenly distributed on the Al_2_O_3_ support. In this study, our focus was on optimizing the carbon yield. The successful application of BO to enhance the carbon yield suggests its potential for optimizing other properties, especially the structural characteristics of CNTs, such as crystallinity, diameter, and the number of walls. The optimization of these structural attributes is a key objective for our future research endeavors.

### 3.3. Predictive Performance

**Figure 4:**
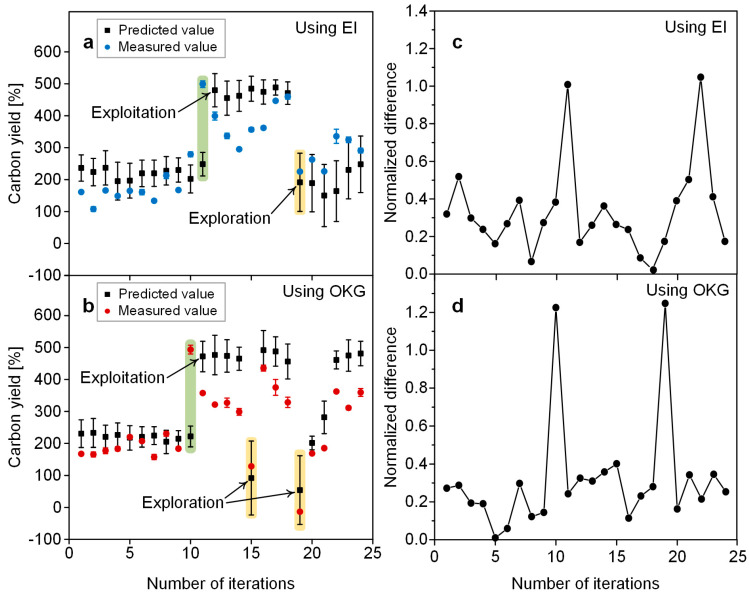
A graph with the predicted and measured values of carbon yield plotted in each iteration when using (**a**) EI and (**b**) OKG. Normalized difference between predicted and measured values of carbon yield in each iteration when using (**c**) EI and (**d**) OKG.

BO can predict the output of a set of input variables recommended by an acquisition function. The predictive performance of BO can be evaluated by comparing the difference between the predicted and measured values in one iteration. The predicted and measured values of the carbon yield in each iteration were plotted in Figure 4a,b, respectively (the corresponding database is available in Tables S1 and S2) and were found to be almost similar. Additionally, for a numerical comparison of the differences between the two values in one iteration, we plotted the normalized differences in Figure 4c,d (the corresponding database is available in Tables S3 and S4). The normalized difference was obtained using the following equation:

Normalized difference = |(predicted value − measured value)/predicted value|
(2)

In most iterations, the normalized difference was low.

However, occasionally, a high normalized difference was observed. When this happened, an interesting behavior was observed. For example, the 11th and 12th iterations when using the EI (Figure 4a) and the 10th and 11th iterations when using the OKG (Figure 4b) are noticeable. In the 11th iteration in Figure 4a and the 10th iteration in Figure 4b, the measured value was significantly higher than the predicted value. When this occurred, both acquisition functions recommended points in the vicinity of the input variable where the measured value increased sharply, demonstrating their exploiting behavior. Meanwhile, they occasionally recommended points with lower predicted values than previously measured values (See the 19th iteration when using the EI and the 15th and 19th iterations when using the OKG). This is the exploratory nature of the acquisition function. As such, in our BO processes, we observed that both acquisition functions struggled to find the global optimum value by balancing between exploring points with high uncertainty and exploiting points with low uncertainty and high predicted value. Both exploration and exploitation play important roles in effectively finding the global optimum, but the two strategies are in a trade-off relationship. Therefore, it is crucial to appropriately adjust the relative intensity between exploration and exploitation.

**Figure 5:**
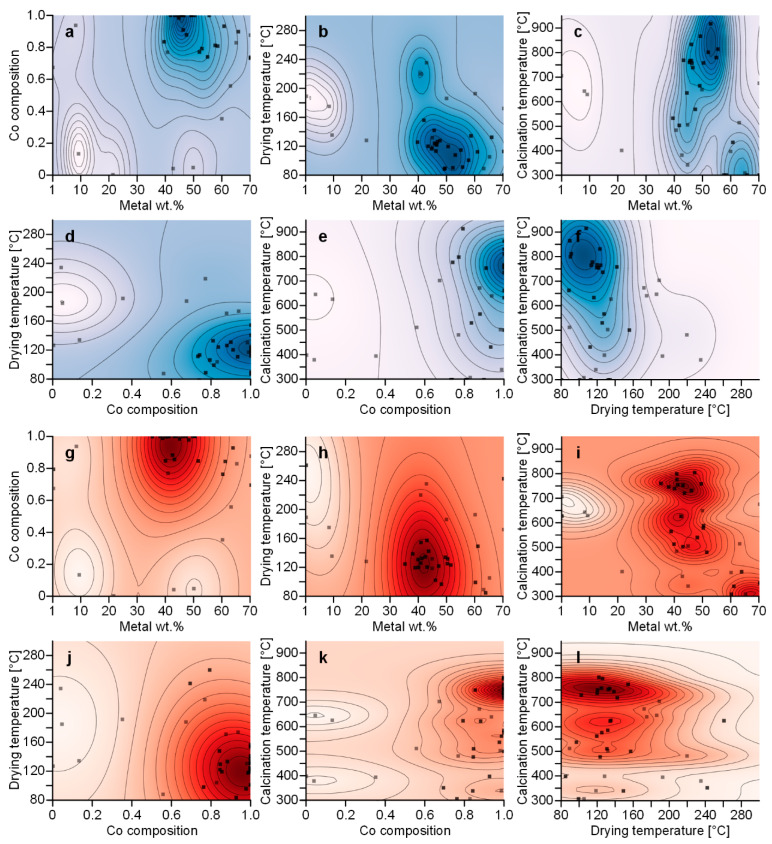
Contour plot predicting carbon yield as a function of two parameters when using (**a**–**f**) EI and (**g**–**l**) OKG, higher chroma indicating a higher carbon yield.

To understand the relationship between the input variables and carbon yield, we visualized contour plots generated by the Gaussian process (Figure 5) (the corresponding database is available in Tables S5 and S6). These contour plots show the predicted carbon yield as a function of two input parameters, with higher chroma indicating a higher carbon yield. The excluded input variables are fixed at the mean of each input parameter. When using the EI (Figure 5a–f) and OKG (Figure 5g–l), the contour plots showed that the maximum carbon yield is predicted to be achieved with a metal weight percent of 40 to 50, a Co composition of 1, a drying temperature of around 120 °C, and a calcination temperature of 700 to 800 °C. A Co composition of 1 means that the addition of Mo to the catalyst has a negative effect on carbon yield. Actually, the finding that the addition of Mo has a negative effect on carbon yield was unexpected. This contradicts the prevailing belief that Mo synergistically interacts with the primary metal catalyst in the CVD synthesis of CNTs. Our study is a data-driven approach and, as such, does not delve into the underlying mechanism. To fully comprehend this behavior, future investigations into the effect of the Mo catalyst in CNT synthesis are needed.

### 3.4. Analysis of As-Synthesized CNTs

**Figure 6:**
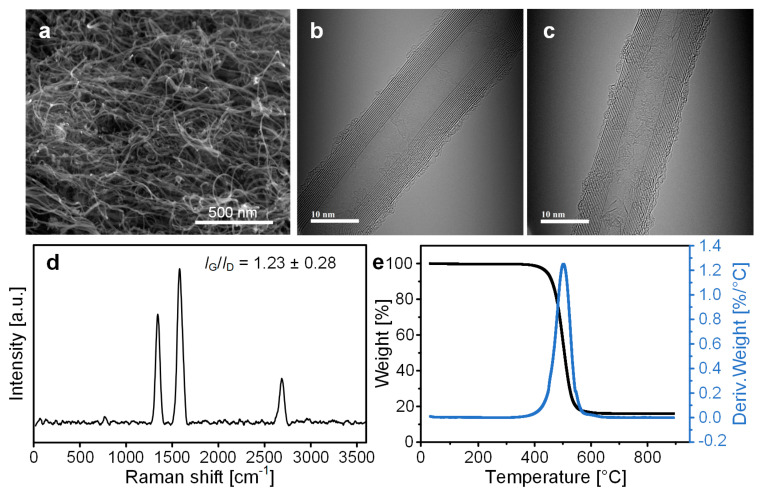
(**a**) SEM image, (**b**,**c**) TEM images, (**d**) Raman spectrum, and (**e**) TGA, DTG data of the typical as-synthesized CNTs.

Figure 6 shows the characterization of typical as-synthesized CNTs using SEM, Raman spectroscopy, and TGA. The SEM image shows that the synthesized CNTs were randomly entangled (Figure 6a). The diameter and number of walls of as-synthesized CNTs were measured using TEM. The diameter was found to be 15.75 ± 5.59 nm, and the number of walls was 14 ± 6 (Figure 6b,c). The corresponding TEM images are available in Figure S2. Raman spectroscopy was employed to confirm the crystallinity of CNTs. The G peak, synonymous with the graphite peak, corresponds to the in-plane vibration of carbon atoms in sp^2^ hybridized carbon structures, typically manifesting around 1580 cm^−1^ in the Raman spectrum. The intensity and position of the G peak can offer insights into the degree of graphitization and the crystallinity of the CNTs. A higher intensity and a narrower peak suggest a higher degree of graphitization and superior crystalline quality. The D peak, commonly known as the defect peak, is linked to the existence of structural defects in the CNTs. These defects may encompass vacancies, edge defects, and other irregularities in the carbon structure. The D peak is usually observed around 1350 cm^−1^. The intensity of the D peak correlates with the density of defects in the CNT structure. A higher intensity of the D peak implies a higher density of defects and is frequently used as an indicator of the structural disorder in the nanotube. The *I*_G_/*I*_D_ ratio represents the level of graphitic structures relative to non-graphitic structures in a carbon sample [44]. The *I*_G_/*I*_D_ ratio of the as-synthesized CNTs was 1.23, indicating a moderate level of graphitic structures (Figure 6d). In general, the crystallinity of the CNT increases with the synthesis temperature. The relatively low quality of our as-synthesized CNTs is attributed to the relatively low synthesis temperature of 690 °C. TGA and DTG were used to assess the purity of our as-synthesized CNTs. The weight loss from 400 to 600 °C was due to the burning of amorphous carbon and CNTs (Figure 6e). Given that carbon materials are expected to decompose at 900 °C, it is reasonable to infer that the residual material is a catalyst composed of Co, Mo, and Al_2_O_3_. The final weight, which is less than 17 wt%, indicates that the synthesized CNTs have high purity. This level of purity is also consistent with the carbon yield of 500%.

## 4. Conclusions

We implemented BO to optimize the preparation of a Co-Mo/Al_2_O_3_ catalyst by wet impregnation for maximizing the carbon yield in a CVD synthesis of CNTs. In the catalyst preparation process, we selected four parameters to optimize: weight percent of metal, ratio of Co and Mo of catalyst, drying temperature, and calcination temperature. To compare the performance of two acquisition functions (EI and OKG), we performed two BO processes in parallel. As a result, both acquisition functions successfully optimized the carbon yield with similar performance, which is attributed to the high robustness of our CNT synthesis system. The results suggest that using the EI with a lower computational load is acceptable if the system has high robustness. Furthermore, we observed that two acquisition functions struggled to find the global optimum value by balancing between exploration and exploitation. The investigation of the contour plots revealed that the addition of Mo has a negative effect on the carbon yield. This study demonstrates the potential of BO in material synthesis, so we strongly recommend BO in optimizing material synthesis as well as catalyst preparation for the CVD synthesis of CNTs.

## Figures and Tables

**Table 1:** Initial database built using Sobol sequence.

Number	Metal wt.%	Co wt.%	Mo wt.%	Drying Temperature [°C]	Calcination Temperature [°C]	Carbon Yield [%]
1	1	1	0	228	829	−11.8 ± 6.0
2	70	61	9	205	789	87.2 ± 16.8
3	50	2	48	224	755	−31.6 ± 3.5
4	9	8	1	209	747	20.2 ± 7.8
5	10	1	9	155	729	4.0 ± 15.7
6	63	35	28	92	579	109.8 ± 7.0
7	45	44	1	150	567	233.7 ± 8.2
8	41	32	9	270	539	159.7 ± 57.3
9	22	0	22	145	431	−13.9 ± 12.1
10	60	21	39	233	426	8.9 ± 15.8
11	43	2	41	291	406	−19.8 ± 4.3
12	45	44	1	132	354	170.6 ± 15.3
13	65	54	11	114	311	244.0 ± 20.5

**Table 2:** Data obtained from a BO process using the EI as an acquisition function.

Number	Metal wt.%	Co wt.%	Mo wt.%	Drying Temperature [°C]	Calcination Temperature [°C]	Carbon Yield [%]
1	61	57	4	123	433	161.0 ± 4.2
2	70	52	18	80	300	107.7 ± 14.8
3	59	48	11	154	300	166.1 ± 8.9
4	42	42	0	183	502	148.8 ± 5.6
5	40	33	7	142	531	164.8 ± 6.8
6	70	51	19	124	300	160.8 ± 16.5
7	58	47	11	108	300	133.6 ± 6.2
8	48	42	6	145	568	212.5 ± 18.1
9	66	59	7	151	300	167.1 ± 6.0
10	45	45	0	129	634	279.1 ± 15.4
11	46	46	0	139	766	499.0 ± 21.1
12	46	46	0	138	832	399.1 ± 24.9
13	46	46	0	164	759	337.0 ± 17.3
14	51	51	0	134	756	295.1 ± 8.1
15	44	44	0	134	767	356.7 ± 12.6
16	47	43	4	136	755	362.0 ± 10.8
17	47	47	0	143	737	446.7 ± 8.9
18	46	46	0	125	766	459.6 ± 15.2
19	49	49	0	91	663	224.9 ± 3.8
20	49	49	0	93	865	262.8 ± 5.7
21	56	56	0	95	813	225.7 ± 4.9
22	52	40	12	93	800	335.7 ± 44.1
23	53	42	11	117	916	324.8 ± 17.6
24	55	41	14	127	779	290.9 ± 14.8

**Table 3:** Data obtained from a BO process using the OKG as an acquisition function.

Number	Metal wt.%	Co wt.%	Mo wt.%	Drying Temperature [°C]	Calcination Temperature [°C]	Carbon Yield [%]
1	64	59	5	86	400	167.8 ± 8.8
2	61	51	10	174	341	165.9 ± 15.4
3	43	43	0	157	501	177.5 ± 18.2
4	52	44	8	123	479	183.2 ± 12.0
5	39	39	0	119	563	219.6 ± 1.9
6	50	50	0	125	578	207.1 ± 7.4
7	60	46	14	99	308	157.7 ± 16.5
8	43	38	5	134	625	229.8 ± 15.3
9	40	34	6	130	512	183.5 ± 5.1
10	41	41	0	132	753	493.6 ± 27.7
11	41	41	0	154	774	357.4 ± 8.5
12	47	47	0	121	803	321.4 ± 6.6
13	36	36	0	124	758	327.2 ± 29.2
14	41	41	0	125	797	298.7 ± 21.1
15	70	49	21	242	353	128.7 ± 3.9
16	46	46	0	102	730	435.7 ± 18.8
17	44	44	0	142	719	375.2 ± 49.4
18	43	37	6	120	751	328.4 ± 32.5
19	1	1	0	260	626	−13.4 ± 3.7
20	50	50	0	131	587	168.8 ± 2.5
21	48	47	1	96	539	185.1 ± 5.9
22	40	40	0	119	738	362.2 ± 8.9
23	50	50	0	133	757	310.1 ± 11.7
24	38	38	0	138	744	359.4 ± 1.2

## Data Availability

Data are contained within the article.

## Supplementary Materials

The following supporting information can be downloaded at: https://www.mdpi.com/article/10.3390/nano14010075/s1, Table S1. Predicted and measured value of carbon yield in each iteration when using EI; Table S2. Predicted and measured value of carbon yield in each iteration when using OKG; Table S3. Normalized difference between predicted and measured value of carbon yield in each iteration when using EI; Table S4. Normalized difference between predicted and measured value of carbon yield in each iteration when using OKG; Table S5. The database used to draw contour plots predicting carbon yield when using EI; Table S6. The database used to draw contour plots predicting carbon yield when using OKG; Figure S1. Energy-dispersive X-ray spectroscopy of Fe catalyst distributed on the Al_2_O_3_; Figure S2. TEM images of as-synthesized CNTs.
