# Supplementary material for: Bayesian Optimization of Wet-Impregnated Co-Mo/Al2O3 Catalyst for Maximizing the Yield of Carbon Nanotube Synthesis
Source: Nanomaterials (Basel). 2023 Dec 26;14(1):75. doi: 10.3390/nano14010075 (PMC10780783; doi:10.3390/nano14010075)
Supplement: Supplementary file 1 [file nanomaterials-14-00075-s001.zip › nanomaterials-2759660-supplementary.pdf]

Supplementary material:

Bayesian optimization of wet-impregnated Co-Mo/Al_2O_3 catalyst for maximizing the yield of carbon nanotube synthesis

Sang Su Shin [1,†], Hyeongyun Song [1,†], Yeon Su Shin [1], Jaegeun Lee [1,2,*], and Tae Hoon Seo [3,*]

[1] School of Chemical Engineering, Pusan National University, Busandaehak-ro 63beon-gil, Geumjeoung-gu, Busan 46241, Republic of Korea

[2] Department of Organic Material Science and Engineering, Pusan National University, Busandaehak-ro 63beon-gil, Geumjeoung-gu, Busan 46241, Republic of Korea

[3] Green Energy and Nano Technology & R&D Group, Korea Institute of Industrial Technology (KITECH), Gwangju 61012, Republic of Korea

[†] These authors contributed equally to this work.

[*] Corresponding authors: jglee@pusan.ac.kr (Jaegeun Lee), thseo@kitech.re.kr (Tae Hoon Seo)

List of Supplementary Table and Figure Captions

Table S1. Predicted and measured value of carbon yield in each iteration when using EI

Table S2. Predicted and measured value of carbon yield in each iteration when using OKG

Table S3. Normalized difference between predicted and measured value of carbon yield in each iteration when using EI

Table S4. Normalized difference between predicted and measured value of carbon yield in each iteration when using OKG

Table S5. Database used to draw contour plot predicting carbon yield when using EI

Table S6. Database used to draw contour plot predicting carbon yield when using OKG

Figure S1. EDS analysis of Fe catalyst distributed on the Al_2O_3.

Figure S2. TEM image of as-synthesized CNTs.

Table S1. Predicted and measured value of carbon yield in each iteration when using EI

Iteration number	Metal wt.%	Co wt.%	Mo wt.%	Drying temperature [°C]	Calcination temperature [°C]	Predicted value of carbon yield [%]	Carbon yield [%]
1	61	57	4	123	433	236.4	161.0 ± 4.2
2	70	52	18	80	300	223.6	107.7 ± 14.8
3	59	48	11	154	300	236.8	166.1 ± 8.9
4	42	42	0	183	502	195.2	148.8 ± 5.6
5	40	33	7	142	531	196.6	164.8 ± 6.8
6	70	51	19	124	300	219.6	160.8 ± 16.5
7	58	47	11	108	300	219.9	133.6 ± 6.2
8	48	42	6	145	568	227.6	212.5 ± 18.1
9	66	59	7	151	300	230.0	167.1 ± 6.0
10	45	45	0	129	634	201.9	279.1 ± 15.4
11	46	46	0	139	766	248.4	499.0 ± 21.1
12	46	46	0	138	832	479.9	399.1 ± 24.9
13	46	46	0	164	759	455.1	337.0 ± 17.3
14	51	51	0	134	756	462.4	295.1 ± 8.1
15	44	44	0	134	767	484.7	356.7 ± 12.6
16	47	43	4	136	755	474.4	362.0 ± 10.8
17	47	47	0	143	737	488.7	446.7 ± 8.9
18	46	46	0	125	766	471.0	459.6 ± 15.2
19	49	49	0	91	663	191.6	224.9 ± 3.8
20	49	49	0	93	865	189.1	262.8 ± 5.7
21	56	56	0	95	813	150.2	225.7 ± 4.9
22	52	40	12	93	800	163.9	335.7 ± 44.1
23	53	42	11	117	916	230.2	324.8 ± 17.6
24	55	41	14	127	779	247.9	290.9 ± 14.8

Table S2. Predicted and measured value of carbon yield in each iteration when using OKG

Iteration number	Metal wt.%	Co wt.%	Mo wt.%	Drying temperature [°C]	Calcination temperature [°C]	Predicted value of carbon yield [%]	Carbon yield [%]
1	64	59	5	86	400	230.5	167.8 ± 8.8
2	61	51	10	174	341	232.8	165.9 ± 15.4
3	43	43	0	157	501	220.0	177.5 ± 18.2
4	52	44	8	123	479	226.3	183.2 ± 12.0
5	39	39	0	119	563	217.5	219.6 ± 1.9
6	50	50	0	125	578	220.1	207.1 ± 7.4
7	60	46	14	99	308	224.3	157.7 ± 16.5
8	43	38	5	134	625	204.8	229.8 ± 15.3
9	40	34	6	130	512	214.4	183.5 ± 5.1
10	41	41	0	132	753	221.8	493.6 ± 27.7
11	41	41	0	154	774	472.0	357.4 ± 8.5
12	47	47	0	121	803	476.6	321.4 ± 6.6
13	36	36	0	124	758	473.8	327.2 ± 29.2
14	41	41	0	125	797	465.0	298.7 ± 21.1
15	70	49	21	242	353	91.8	128.7 ± 3.9
16	46	46	0	102	730	491.8	435.7 ± 18.8
17	44	44	0	142	719	487.8	375.2 ± 49.4
18	43	37	6	120	751	456.2	328.4 ± 32.5
19	1	1	0	260	626	54.2	-13.4 ± 3.7
20	50	50	0	131	587	201.4	168.8 ± 2.5
21	48	47	1	96	539	281.5	185.1 ± 5.9
22	40	40	0	119	738	461.2	362.2 ± 8.9
23	50	50	0	133	757	474.9	310.1 ± 11.7
24	38	38	0	138	744	481.2	359.4 ± 1.2

Table S3. Normalized difference between predicted and measured value of carbon yield in each iteration when using EI.

Iteration number	Predicted value of carbon yield [%]	Carbon yield [%]	Normalized difference
1	236.4	161.0 ± 4.2	0.32
2	223.6	107.7 ± 14.8	0.52
3	236.8	166.1 ± 8.9	0.30
4	195.2	148.8 ± 5.6	0.24
5	196.6	164.8 ± 6.8	0.16
6	219.6	160.8 ± 16.5	0.27
7	219.9	133.6 ± 6.2	0.39
8	227.6	212.5 ± 18.1	0.07
9	230.0	167.1 ± 6.0	0.27
10	201.9	279.1 ± 15.4	0.38
11	248.4	499.0 ± 21.1	1.01
12	479.9	399.1 ± 24.9	0.17
13	455.1	337.0 ± 17.3	0.26
14	462.4	295.1 ± 8.1	0.36
15	484.7	356.7 ± 12.6	0.26
16	474.4	362.0 ± 10.8	0.24
17	488.7	446.7 ± 8.9	0.09
18	471.0	459.6 ± 15.2	0.02
19	191.6	224.9 ± 3.8	0.17
20	189.1	262.8 ± 5.7	0.39
21	150.2	225.7 ± 4.9	0.50
22	163.9	335.7 ± 44.1	1.05
23	230.2	324.8 ± 17.6	0.41
24	247.9	290.9 ± 14.8	0.17

Table S4. Normalized difference between predicted and measured value of carbon yield in each iteration when using OKG.

Iteration number	Predicted value of carbon yield [%]	Carbon yield [%]	Normalized difference
1	230.5	167.8 ± 8.8	0.27
2	232.8	165.9 ± 15.4	0.29
3	220.0	177.5 ± 18.2	0.19
4	226.3	183.2 ± 12.0	0.19
5	217.5	219.6 ± 1.9	0.01
6	220.1	207.1 ± 7.4	0.06
7	224.3	157.7 ± 16.5	0.30
8	204.8	229.8 ± 15.3	0.12
9	214.4	183.5 ± 5.1	0.14
10	221.8	493.6 ± 27.7	1.22
11	472.0	357.4 ± 8.5	0.24
12	476.6	321.4 ± 6.6	0.33
13	473.8	327.2 ± 29.2	0.31
14	465.0	298.7 ± 21.1	0.36
15	91.8	128.7 ± 3.9	0.40
16	491.8	435.7 ± 18.8	0.11
17	487.8	375.2 ± 49.4	0.23
18	456.2	328.4 ± 32.5	0.28
19	54.2	-13.4 ± 3.7	1.25
20	201.4	168.8 ± 2.5	0.16
21	281.5	185.1 ± 5.9	0.34
22	461.2	362.2 ± 8.9	0.21
23	474.9	310.1 ± 11.7	0.35
24	481.2	359.4 ± 1.2	0.25

Table S5. Database used to draw contour plot predicting carbon yield when using EI

Number	Metal wt.%	Co wt.%	Mo wt.%	Drying temperature [°C]	Calcination temperature [°C]	Carbon yield [%]
1	1	1	0	228	829	-11.8 ± 6.0
2	70	61	9	205	789	87.2 ± 16.8
3	50	2	48	224	755	-31.6 ± 3.5
4	9	8	1	209	747	20.2 ± 7.8
5	10	1	9	155	729	4.0 ± 15.7
6	63	35	28	92	579	109.8 ± 7.0
7	45	44	1	150	567	233.7 ± 8.2
8	41	32	9	270	539	159.7 ± 57.3
9	22	0	22	145	431	-13.9 ± 12.1
10	60	21	39	233	426	8.9 ± 15.8
11	43	2	41	291	406	-19.8 ± 4.3
12	45	44	1	132	354	170.6 ± 15.3
13	65	54	11	114	311	244.0 ± 20.5
14	61	57	4	123	433	161.0 ± 4.2
15	70	52	18	80	300	107.7 ± 14.8
16	59	48	11	154	300	166.1 ± 8.9
17	42	42	0	183	502	148.8 ± 5.6
18	40	33	7	142	531	164.8 ± 6.8
19	70	51	19	124	300	160.8 ± 16.5
20	58	47	11	108	300	133.6 ± 6.2
21	48	42	6	145	568	212.5 ± 18.1
22	66	59	7	151	300	167.1 ± 6.0
23	45	45	0	129	634	279.1 ± 15.4
24	46	46	0	139	766	499.0 ± 21.1
25	46	46	0	138	832	399.1 ± 24.9
26	46	46	0	164	759	337.0 ± 17.3
27	51	51	0	134	756	295.1 ± 8.1
28	44	44	0	134	767	356.7 ± 12.6
29	47	43	4	136	755	362.0 ± 10.8
30	47	47	0	143	737	446.7 ± 8.9
31	46	46	0	125	766	459.6 ± 15.2
32	49	49	0	91	663	224.9 ± 3.8
33	49	49	0	93	865	262.8 ± 5.7
34	56	56	0	95	813	225.7 ± 4.9
35	52	40	12	93	800	335.7 ± 44.1
36	53	42	11	117	916	324.8 ± 17.6
37	55	41	14	127	779	290.9 ± 14.8

Table S6. Database used to draw contour plot predicting carbon yield when using OKG

Number	Metal wt.%	Co wt.%	Mo wt.%	Drying temperature [°C]	Calcination temperature [°C]	Carbon yield [%]
1	1	1	0	228	829	-11.8 ± 6.0
2	70	61	9	205	789	87.2 ± 16.8
3	50	2	48	224	755	-31.6 ± 3.5
4	9	8	1	209	747	20.2 ± 7.8
5	10	1	9	155	729	4.0 ± 15.7
6	63	35	28	92	579	109.8 ± 7.0
7	45	44	1	150	567	233.7 ± 8.2
8	41	32	9	270	539	159.7 ± 57.3
9	22	0	22	145	431	-13.9 ± 12.1
10	60	21	39	233	426	8.9 ± 15.8
11	43	2	41	291	406	-19.8 ± 4.3
12	45	44	1	132	354	170.6 ± 15.3
13	65	54	11	114	311	244.0 ± 20.5
14	64	59	5	86	400	167.8 ± 8.8
15	61	51	10	174	341	165.9 ± 15.4
16	43	43	0	157	501	177.5 ± 18.2
17	52	44	8	123	479	183.2 ± 12.0
18	39	39	0	119	563	219.6 ± 1.9
19	50	50	0	125	578	207.1 ± 7.4
20	60	46	14	99	308	157.7 ± 16.5
21	43	38	5	134	625	229.8 ± 15.3
22	40	34	6	130	512	183.5 ± 5.1
23	41	41	0	132	753	493.6 ± 27.7
24	41	41	0	154	774	357.4 ± 8.5
25	47	47	0	121	803	321.4 ± 6.6
26	36	36	0	124	758	327.2 ± 29.2
27	41	41	0	125	797	298.7 ± 21.1
28	70	49	21	242	353	128.7 ± 3.9
29	46	46	0	102	730	435.7 ± 18.8
30	44	44	0	142	719	375.2 ± 49.4
31	43	37	6	120	751	328.4 ± 32.5
32	1	1	0	260	626	-13.4 ± 3.7
33	50	50	0	131	587	168.8 ± 2.5
34	48	47	1	96	539	185.1 ± 5.9
35	40	40	0	119	738	362.2 ± 8.9
36	50	50	0	133	757	310.1 ± 11.7
37	38	38	0	138	744	359.4 ± 1.2

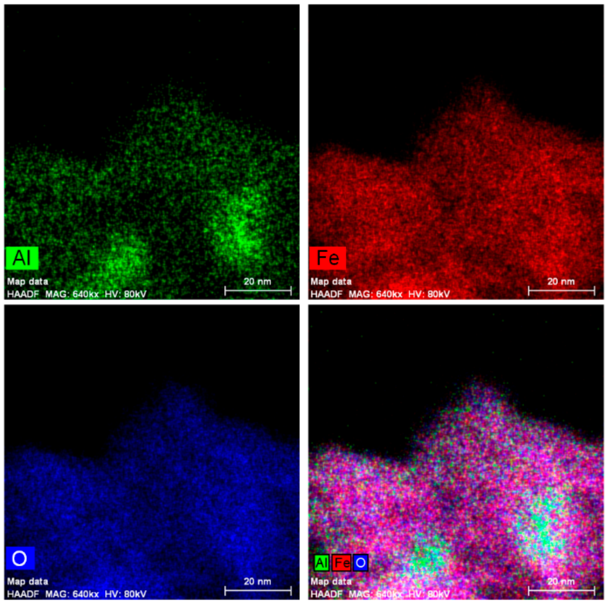

Figure S1. Energy-dispersive X-ray spectroscopy of Fe catalyst distributed on the Al_2O_3.

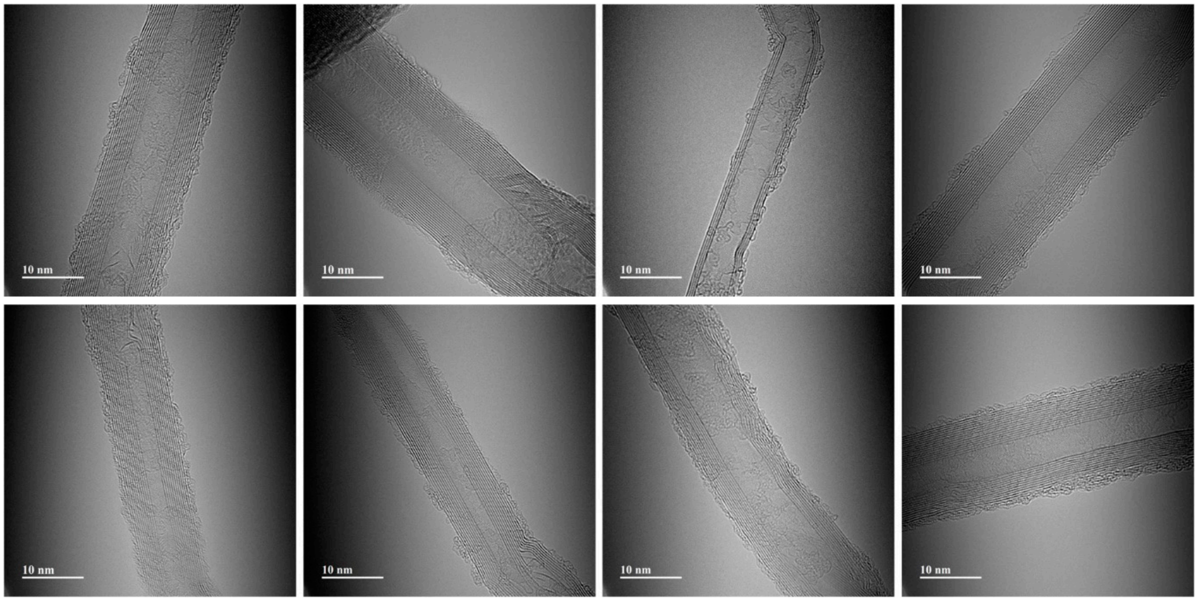

Figure S2. TEM image of as-synthesized CNTs.
